# Reply to Jingu, K. Comment on “Mitamura et al. Treatment Strategies for Locoregional Recurrence in Esophageal Squamous-Cell Carcinoma: An Updated Review. *Cancers* 2024, *16*, 2539”

**DOI:** 10.3390/cancers17203334

**Published:** 2025-10-16

**Authors:** Atsushi Mitamura, Shingo Tsujinaka, Toru Nakano, Kentaro Sawada, Chikashi Shibata

**Affiliations:** Division of Gastroenterologic Surgery, Department of Surgery, Tohoku Medical and Pharmaceutical University, Sendai, Miyagi 983-8536, Japan; sher_lock78@yahoo.co.jp (A.M.); nakano@hosp.tohoku-mpu.ac.jp (T.N.); k.s.zeppelin.0314@gmail.com (K.S.); cshibata@tohoku-mpu.ac.jp (C.S.)

We express our sincere appreciation for your thoughtful and constructive commentary [1] on our article. Your insights contribute meaningfully to the current academic discussion and promote further understanding of unresolved challenges in the treatment of locoregional recurrence in esophageal squamous cell carcinoma.

Chen et al. [2] conducted a Phase II clinical trial to evaluate the efficacy of weekly treatment of 5-florouracil (5-FU) and cisplatin concurrent with radiotherapy for patients with locoregionally recurrent esophageal squamous-cell carcinoma (ESCC). Forty-eight patients were enrolled, with median progression-free survival (PFS) and overall survival (OS) durations of 14 and 27 months, respectively. No treatment-related Grade 4 adverse events were observed; thus, the authors’ aim to reduce toxicity while maintaining favorable therapeutic outcomes was achieved. Although the study was not cited in the 2022 esophageal cancer practice guidelines [3,4], we believe its findings underscore the potential of concurrent chemoradiotherapy (CCRT) as a viable alternative to standard chemotherapy, using fluorouracil combined with cisplatin (FP).

Jingu et al. [5] reported the long-term results of their Phase II study on locoregionally recurrent ESCC treatment, in which nedaplatin was utilized as a substitute for cisplatin in combination with 5-FU, and the 5-year median PFS and OS were 25 months and 21 months, respectively. Compared to cisplatin, nedaplatin is associated with reduced nephrotoxicity and gastrointestinal toxicity, potentially allowing for long-term administration in elderly patients or those with impaired renal function. Therefore, nedaplatin has been recognized as a viable alternative for patients who are intolerant to cisplatin due to impaired renal and/or cardiac function [3].

We believe that future literature reviews or clinical guidelines should consider including these studies [2,5] as evidence supporting CCRT as an effective treatment option for locoregional recurrence in ESCC. However, the results must be carefully interpreted due to the Phase II nature of these studies, including their single-arm designs, small sample sizes, and limited patient inclusion.

In another study, Jingu et al. [6] reported the long-term outcomes of platinum-based CCRT in 35 ESCC patients who had undergone curative esophagectomy with two- or three-field lymph node dissection and subsequently developed lymph node metastases. The 3- and 5-year OS rates were 50.6% and 39.2%, respectively, with a median survival duration of 39 months. Early treatment-related adverse events included Grade 3 hematologic toxicity in twelve cases and one case of non-hematologic toxicity; however, no Grade 3 or higher adverse events were observed in the late phase. Based on these findings, the authors concluded that the platinum-based CCRT was highly effective. Among the 35 cases, 31 were treated with nedaplatin, which may have contributed to the low adverse event incidence. Regarding prognosis, the authors reported significantly longer survival in patients whose metastasis was confined to the supraclavicular lymph nodes, as compared to those with metastasis in the mediastinum or abdominal lymph nodes. In addition, a correlation was observed between metastatic lymph node size and survival, with cervical lymph nodes generally smaller than those in other regions at the time of diagnosis; these findings suggest the need for evaluating whether CCRT alone provides sufficient therapeutic efficacy for solitary lymph node metastasis after curative ESCC resection. The study’s limitations include its non-randomized, retrospective design and small sample size; furthermore, the observed optimistic results may be primarily applicable to solitary lymph node metastasis, but not to patients with concurrent metastases at multiple sites. Multi-center studies with larger patient cohorts are essential to establish more robust treatment strategies.

While some comments noted that the interpretation of the CheckMate 648 trial may have been insufficiently addressed, the main report by Doki et al. [7], which we cited in the “Chemotherapy” section, presented the oncologic outcomes based on overall disease status at trial entry (metastatic, recurrent—locoregional, recurrent—distant, and unresectable—advanced); however, it did not provide detailed analyses for each individual subgroup. Additionally, Table 1 in our manuscript was designed to compare and summarize results across multiple clinical trials; because several of these trials also lacked specific data regarding locoregional recurrence alone, the table was formatted accordingly to maintain consistency across all referenced studies. Nevertheless, as highlighted in the comment, the CheckMate 648 trial failed to demonstrate significant survival benefit for patients with locoregional recurrence, with the median OS durations reported as 14.8, 13.9, and 13.5 months in the nivolumab plus chemotherapy group, the nivolumab plus ipilimumab group, and the chemotherapy-only group, respectively (Figure 3SB and 3SD in reference [7]). We appreciate the reminder that the interpretation of findings from clinical trials requires a meticulous review of both the main text and the Supplementary Materials.

In a recent retrospective study, Wu et al. [8] evaluated immunotherapy efficacy with or without concurrent radiotherapy in metastatic or recurrent ESCC patients. Although the study’s results did not demonstrate significant improvement for all patients in median PFS (5.45 months in the radiotherapy group and 4.60 months in the non-radiotherapy group, *p* = 0.660) or OS durations (11.9 months in the radiotherapy group and 10.3 months in the non-radiotherapy group, *p* = 0.890), it showed an improvement in patients with locoregional recurrence in median PFS (11.27 months in radiotherapy group and 4.17 months in non-radiotherapy group, *p* = 0.081) and OS durations (19.48 months in the radiotherapy group and 7.69 months in the non-radiotherapy group, *p* = 0.026). In addition, Zhao et al. [9] retrospectively investigated immune checkpoint inhibitor efficacy with or without concurrent radiotherapy/chemotherapy in locally advanced or recurrent/metastatic ESCC patients. The study’s results demonstrated that the number of ICI cycles, radiotherapy intervention, and dysphagia were independent factors affecting OS (hazard ratio [HR] = 0.39, 2.043, and 0.365, respectively; *p* = 0.018, 0.001, and 0.032, respectively). Immunotherapy combined with radiotherapy or chemotherapy is emerging as promising approach for locally advanced or recurrent ESCC treatment. Despite this potential, the relevant studies are limited by their single-center, retrospective designs, as well as the inherent risks of selection bias, potential publication bias, and the lack of programmed death ligand 1 (PD-L1) expression evaluation during the treatment [8,9]. The current esophageal cancer treatment guidelines have yet to formally recognize this combination therapy as a standard treatment modality [3,4].

When focusing on locoregionally recurrent ESCC, most of the available evidence has been derived from studies with relatively small sample sizes or retrospective designs, which is partly attributable to the disease’s rarity, as well as the frequent inclusion of locoregional recurrence with distant metastases and/or unresectable cases, even in prospective studies involving larger cohorts; furthermore, most of the studies cited in our review that favored surgical outcomes primarily featured retrospective analyses of highly selected patients considered eligible for surgical intervention. Therefore, as the comments pointed out, monotherapies, such as “surgery”, “radiotherapy”, and “immunotherapy”, may possibly be omitted in the future as a result of combination therapy advancement and development; thus, it would be prudent to reassess and revise the suggested algorithm in our review, with additional accumulated evidence.

The future direction for locoregionally recurrent ESCC treatment mandates dedicated and high-level evidence. First, prospective, randomized trials are warranted. The study population should be refined to concentrate on patients with isolated locoregional recurrence, without distant metastases, ensuring a homogeneous cohort for evaluation. Secondly, the integration of novel systemic agents, such as programmed cell death 1 (PD-1) inhibitors, should be thoroughly explored. Finally, future trials should incorporate translational research to refine patient selection through the identification and validation of predictive biomarkers, including PDL-1 expression, circulating tumor DNA kinetics, or specific genetic mutations.

We are grateful for the opportunity to critically assess and discuss key aspects of our review article and look forward to continued engagement with the academic community on these important topics.
